# Photonic Properties of InAs Quantum Dots by Post‐Growth Maskless Site‐Selective Focused Ion Beam Implantation

**DOI:** 10.1002/nap2.70162

**Published:** 2026-06-13

**Authors:** Mehdi Ahmadian, Young In Na, Maddison Coke, Daniel Blight, Akshay Kumar Verma, Mark C. Rosamond, Ian Farrer, Jon Heffernan, Richard J. Curry

**Affiliations:** ^1^ Photon Science Institute (PSI) Department of Electrical & Electronic Engineering University of Manchester Manchester UK; ^2^ EPSRC Centre for Doctoral Training (CDT) in Compound Semiconductor Manufacturing Cardiff University Cardiff UK; ^3^ National Epitaxy Facility (NEF) Nanoscience and Technology Centre University of Sheffield Sheffield UK; ^4^ School of Electronic and Electrical Engineering University of Leeds Leeds UK

**Keywords:** FIB, III‐V semiconductors, intermixing, ion implantation, photonic integrated circuit (PIC), quantum dot (QD)

## Abstract

We report the maskless site‐selective modification of InAs quantum dot (QD) photonic properties via focused ion beam (FIB) implantation of antimony (Sb, group V), controlling emission wavelength and linewidth. Contrasting postgrowth methods yielding emission blueshift, our approach achieves bidirectional (blue and red) emission wavelength shifts, offering flexibility, without requiring an implantation mask for site selectivity. Our experimental results also address inhomogeneous broadening due to size and spatial variations in epitaxial QDs enhancing spectral uniformity. Additionally, focused ion implantation suppresses wetting layer (WL) emission, improving spectral purity, with controlled PL reduction tailored by ion dose. The wider ability to achieve red shifts enhanced linewidth and reduced WL emission without using postimplantation thermal annealing through this method is confirmed using boron (B, group III). Selected for its contrasting electronic doping and significantly different mass compared with Sb, consistent spectral modification was achieved. Dynamic and static simulations were carried out to determine ion and defect profiles, and intermixing corroborated by high‐resolution micro‐PL spectroscopy resolving individual QD emission. This scalable approach supports site‐selective QD engineering for advanced photonic integrated circuits (PICs), nanophotonic, and quantum systems such as single‐photon emitters and solid‐state quantum memories.

## Graphical abstract

A low‐energy focused ion beam process achieves localized spectral modification of InAs quantum dots. By leveraging low ion straggle and a compact damage volume, this methodology offers unique spatial control and accuracy. This maskless approach provides a scalable platform for site‐selective engineering for advanced photonic integrated circuits and quantum systems.

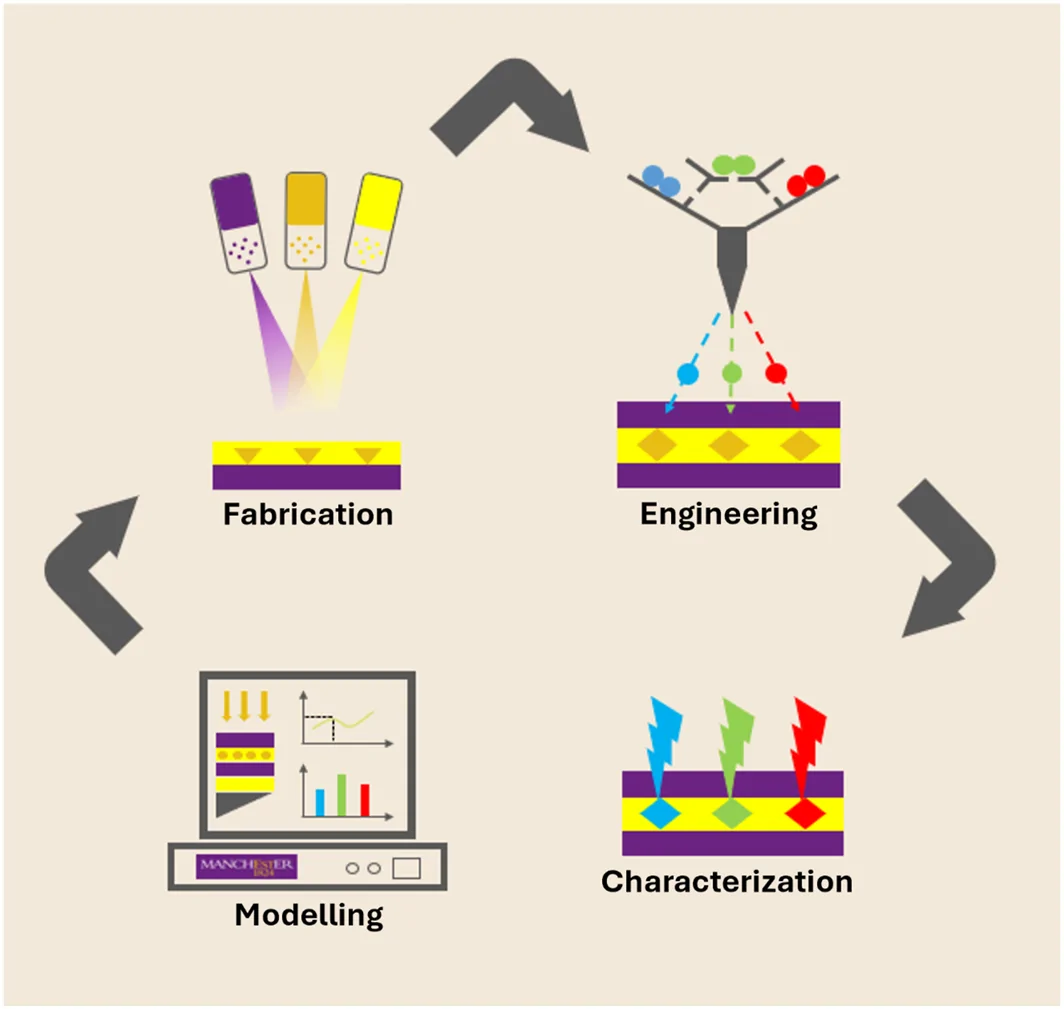

## 1 Introduction

Photonic integrated circuits (PICs) offer substantial advantages in terms of compact size, low‐loss optical properties, scalability, and functional versatility, making them attractive for diverse applications including optical communication [1], light detection and ranging (LiDAR) [2], sensing [3], and quantum computing [4]. A key challenge in advancing PIC technologies is the ability to spatially tailor and locally control photonic properties, particularly for active components such as quantum dot (QD) emitters [5, 6].

Although methods, such as selective‐area epitaxial regrowth, can achieve spatial variation in emission wavelength, they are inherently complex, limited to early fabrication stages, and often lack the flexibility and scalability required for large‐scale integration [7, 8]. In contrast, postgrowth techniques allow modification of material or optical properties after fabrication, offering compatibility with completed devices and enabling application‐specific tuning. Such approaches are especially promising for monolithic PIC platforms where active and passive elements must be integrated and individually engineered.

As the need for efficient compact PICs grows, QDs have gained attention due to their distinctive properties due to their discrete energy levels [9, 10, 11] and potential for scalable integration in such systems [12, 13]. These properties, combined with enhanced photon generation rates and strong tolerance to defects, make QDs highly appealing for photonic circuits [14]. III–V epitaxial QDs, particularly InAs, have gained widespread interest due to their favorable optical properties and compatibility with commonly used compound semiconductor substrates, such as GaAs and InP, via standard epitaxial techniques such as the Stranski–Krastanow (S–K) method. However, one limitation of the S–K method is the inhomogeneous broadening that is caused by variation in QD size, shape and spatial distribution, which reduces the spectral purity of the emission and restricts the potential of QDs in applications requiring narrow linewidths [15, 16, 17].

To engineer the properties and mitigate these issues, significant efforts have been made to refine growth techniques aimed at reducing inhomogeneous broadening and achieving better photonic performance [18, 19, 20]. As a result, InAs QDs have demonstrated considerable progress, particularly in O‐/C‐band laser devices [21, 22, 23]. Furthermore, expanding the functionality of these QDs to shorter wavelengths, such as 880 and 980 nm, is of interest for emerging applications including solid‐state quantum memory and quantum information processing [24, 25].

However, despite these advancements, the ability to modify the photonic properties of site‐specific QDs remains a key challenge. It is especially the case for quantum photonic applications where single‐photon sources and quantum memories require high spectral uniformity and spatial control. Although extensive research has focused on optimizing QD growth techniques, reports on their post‐growth modification remain relatively limited. This gap is primarily due to the challenges associated with maintaining quality and precisely spatially selective modification of the properties of epitaxial QDs after their initial growth stage. Nevertheless, a robust site‐selective postgrowth tailoring strategy is indispensable for advancing quantum technologies, enabling the modification of photonic properties without being constrained by the growth process itself.

Post‐growth approaches are generally categorized into two main groups: impurity‐free vacancy disordering (IFVD) and damage‐enhanced modification methods. Although IFVD methods are straightforward and relatively simple [26, 27], they suffer from several issues that are especially critical for PIC and quantum technologies including strong dependency on inherent defects, limited selectivity, and poor reproducibility [28].

In contrast, ion implantation, as a defect‐engineering technique, offers unique spatial selectivity both laterally and vertically, making it a powerful tool for engineering semiconductor materials. It enables flexible material modification through several tunable parameters, including ion species, ion energy, implantation temperature, and ion dose. This versatility makes ion implantation attractive for the modification of material properties in a controlled and scalable way. However, conventional ion implantation approaches also present several limitations, most notably the need for physical implantation masks due to the micrometer (or larger) ion beam diameters used. These constraints lead to reduced spatial accuracy, limited selectivity, and additional fabrication complexity. As a result, although traditional ion implantation is useful for modifying ensembles of QDs, its precision is insufficient for engineering localized or individual QDs, which are appealing for quantum photonic applications [29, 30].

To overcome the limitations of conventional implantation for localized engineering, a precise and reliable approach is crucial. Recent advances in focused ion beam (FIB) implantation have started to emerge as a promising alternative, offering high spatial resolution and flexibility without the need for physical masks [31]. This technique enables deterministically controlled ion delivery with nanoscale spatial accuracy, addressing the key challenges of selectivity and scalability, simultaneously. Although FIB implantation has previously been employed for substrate patterning to create site‐controlled QD arrays [32, 33], its potential for direct postgrowth modification of localized III–V QDs remains largely unexplored. In this work, we demonstrate a maskless, site‐selective, and fully postgrowth approach for modifying the photonic properties of InAs QDs using FIB implantation, highlighting its promise for precise and scalable modification of components in PICs.

Previous studies employing either IFVD or damage‐enhanced techniques have predominantly reported blueshifts in QD ensemble emission spectra following modification. In contrast, we demonstrate that a redshift can be achieved in InAs QDs through a single‐step FIB implantation process without the need for any subsequent thermal annealing. Furthermore, we show that a controlled blueshift is also attainable by following the implantation with a rapid thermal annealing (RTA) step. Notably, this behavior is consistent across both heavy and light ion species from groups III and V, supporting the generality and versatility of this post‐growth modification approach. Together, these findings open new opportunities for the selective tuning of QD optical properties for integrated photonic and quantum technologies.

## 2 Material Growth

The samples were grown by solid source molecular beam epitaxy (MBE) on semi‐insulating GaAs(100) wafers. The single QD layer were grown in a standard heterostructure designed for potential ion implantation (Figure 1a). The following layer sequence was adopted for the growth of the QD samples: first a 300 nm GaAs buffer layer was grown at 590°C after thermal oxide desorption at 600°C, followed by a 50 nm Al_0.3_Ga_0.7_ As barrier layer. Subsequently, the substrate temperature was ramped down to 480°C over a period of 6 min and stabilized for approximately 2 min to ensure a consistent growth temperature of 480 ± 1°C, as monitored by a kSA BandiT band‐edge thermometry system. Then 5 nm of GaAs layer was deposited followed by InAs deposition under a constant As_2_ overpressure of 3.71E‐06 Torr. A slow InAs deposition rate of 0.011 ML s^−1^ was employed with continuous substrate rotation at 10 rpm, and a QD density (∼1 QD μm^−2^) was achieved by adjusting the total amount of Indium deposited. After the InAs deposition, the sample was annealed at the growth temperature for 15 s after which the QDs were partially capped with a 5 nm GaAs layer. Subsequently, the substrate temperature was ramped to 600°C (∼20°C/min), followed by deposition of a 20 nm Al_0.3_Ga_0.7_ As upper barrier and a final 20 nm GaAs cap layer.

**FIGURE 1:**
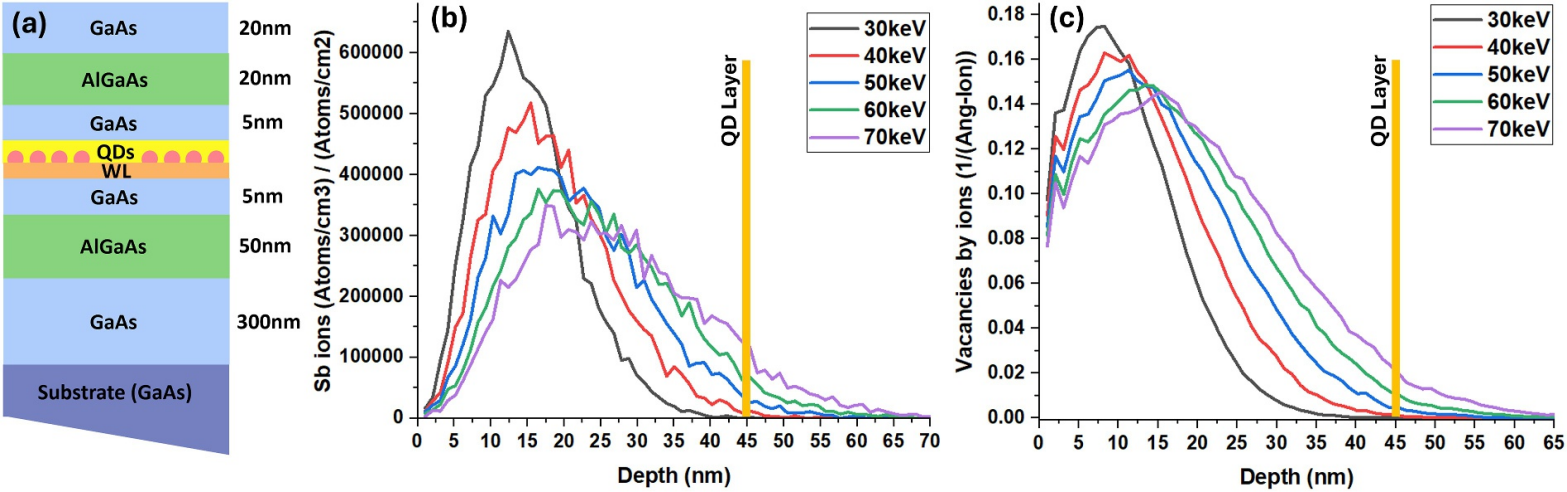
Schematic illustration of grown InAs QD structure (a), ion implantation simulation of Sb ion range (b) and induced vacancies (c) within structure as a function of ion energy.

## 3 Post‐Growth Engineering

Following growth of high‐quality InAs QDs, designing a functional implantation strategy is the most crucial factor. This is especially critical when working with heavier ion species since they can create considerable defect clusters within such nanostructures that can destroy QDs or reduce their optical quality significantly. On the other hand, if the ions are implanted far from the dot layer, no change occurs in QD properties after the process.

The QD wafer was cleaved into 4 mm × 4 mm chips. Markers were created on each chip using photolithography and etching (Figure 2a‐inset) to enable reproducible location of QDs in specific regions. Low‐temperature high‐resolution (∼5 μm spot diameter) micro‐PL (μPL) spectroscopy (635 nm excitation, 20 μW power) was used to characterize the sample prior to further processing, providing detection of individual *δ*‐like emission from single QDs. The spectra were obtained using an Acton SpectraPro SP‐2750 spectrometer and a liquid nitrogen cooled PyLoN Si CCD camera. Based on the spectrometer dispersion and the entrance slit width, the spectral resolution is ∼0.02 nm. Rigorous data selection was implemented to ensure that the reported spectra only represent stable single QD emissions. Individual peaks were validated based on applying a signal‐to‐noise intensity threshold of 150 counts. Power‐dependent micro‐PL measurements further confirmed the linear and super‐linear scaling of these features, identifying them as neutral and charged excitonic states rather than stochastic background fluctuations [34]. The InAs/GaAs QD wafers utilized in this study were grown at the National Epitaxy Facility (NEF) using mature MBE protocols. This material platform has been extensively validated over decades for its quantum photonic integrity and predictable carrier dynamics [35, 36].

**FIGURE 2:**
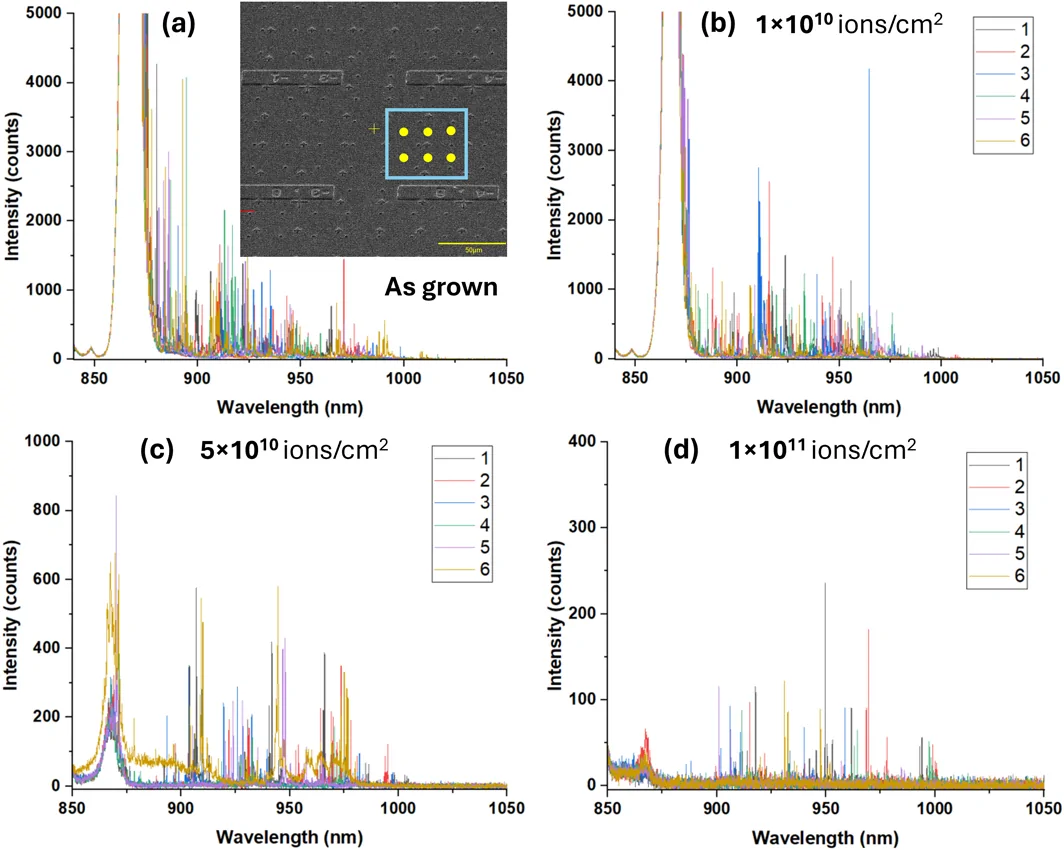
635 nm excited μPL spectra from InAs QDs obtained at 5 K for (a) as‐grown QDs, (b)–(d) 50 keV Sb implanted with doses of 1E10 ions/cm^2^, 5E10 ions/cm^2^, and 1E11 ions/cm^2^, respectively. Each panel consists of six μPL spectra taken from each region numbered 1–6. Inset: exemplar six μPL spots (yellow circles) and implanted regions (blue frame) are shown in SEM image. The scale bar in the inset is 50 μm.

Following the postgrowth, preimplantation μPL spectroscopy samples were transferred to the FIB implantation chamber. The FIB system used in these studies, the Platform for Nanoscale Advanced Materials Engineering (P‐NAME) (Q‐One, Ionoptika) [31] is equipped with an integrated high‐resolution scanning electron microscope (SEM) that allows precise location of regions for implantation using the co‐incident ion beam. Using this capability defined regions (each 50 mm × 50 mm) within a single sample were implanted with Sb ions (at a 3° angle of incidence from the normal to the surface to mitigate channeling) with doses of 1E10, 5E10, 5E11, and 1E12 ions/cm^2^ and with B ions with doses of 1E10, 1E11, 1E12, and 1E13 ions/cm^2^. The Sb ion energy was 50 keV (obtained using Sb^2+^ ions with 25 kV acceleration) and a B ion energy of 10 keV chosen based upon the modeling (see Figure 1b,c and Supporting Information S1: Figure S1) and available beam energy values of the FIB implantation facility.

Postimplantation characterization was carried out by repeating μPL spectroscopy in the same regions as for the preimplantation measurements. This was followed by a series of RTAs (JIPELEC JetFirst 100) under a nitrogen atmosphere and GaAs proximity cap for 30 s at the temperatures described within the following section. The RTA duration was fixed at 30 s aimed at achieving reliable vacancy migration whilst preventing thermal surface degradation based on prior work [37, 38, 39]. This provides a compromise that ensures sufficient time for vacancies to reach the QD interface to trigger interdiffusion without inducing excessive thermal broadening of the nonimplanted regions. Further μPL spectroscopy characterization was performed after each stage.

## 4 Results and Discussion

To introduce ions into the QDs, a suitable implantation energy must be used. However, it is also required that ion damage to the underlying structure and QDs is minimized. Modeling was performed using SRIM [40] to obtain the Sb ion depth (range), lateral spread (straggle) and the vacancy concentration (damage) as a function of ion energy for the structure shown in Figure 1a. Figure 1b,c show the ion range and vacancy damage, respectively, as a function of energy. It is clear that aiming to place the implanted Sb ions with an average depth similar to the QDs (45 nm) would require high (> 75 keV) energy and result in significant damage to the structure and QDs. Although this damage can be used to promote the intermixing mechanism, at the levels predicted for high energies it may significantly degrade the optical properties of the QDs. QDs are embedded at that depth beneath the surface to mitigate the effects of surface states and associated non‐radiative recombination pathways, thereby preserving the radiative efficiency and optical quality.

A further consideration is the ion doping concentration within the QDs that is desired. For the low levels desired, we are able to rely on using the end of range tail to dope the QDs. For example, a doping concentration of 1E12 ions/cm^2^ is equivalent to ∼20 ions within a 50 nm diameter (similar to a QD size) where less than one Sb ion (on average) stops in range of a QD. Inspection of Figure 1b shows that it is possible to introduce Sb ions into the QD layer using the end‐of‐range tail at energies as low as 40 keV. Similarly, Figure 1c shows the damage profile as a function of ion energy including the QD layer. To balance the desire to introduce modest damage (to aid intermixing) and to deliver sufficient Sb ions in proximity to the QD layer, we selected an ion energy of 50 keV for these studies.

A final consideration is the ion induced sputtering of the surface which will occur during implantation. Dynamic modeling using TRIDYN [41] was performed for a series of 50 keV Sb ion doses to understand at what dose sputtering starts to impact on the doping profile. It was found for ion doses below 1E13 ions/cm^2^ negligible surface recession occurs, whereas for doses above 1E14 ions/cm^2^, this should be factored into modeling of the final ion range distribution (Supporting Information S1: Figure S2). Our experimental results, presented within, find that for doses of 1E12 ions/cm^2^ damage to the QD layer quenches all emission which cannot be recovered via annealing. Therefore, for the doses utilized for the optical studies, surface sputtering during implantation does not need to be considered.

μPL measurements were carried out at 5K with an excitation spot diameter of ∼5 μm typically covering a few dozen dots. Figure 2 shows the results of μPL spectroscopy for as‐grown and the 50 keV Sb‐implanted QDs (as implanted) with each graph showing a set of spectra. The appearance of multiple emission lines in the μPL spectra (exceeding what would be expected for a single μPL spot at a QD density of the sample) is due to graph displaying the spectrum recorded from six different μPL sampling coordinates across the implanted region to ensure statistical representation.

Figure 2a shows the PL spectra from the as‐grown QDs, displaying broad emission band at ∼870 nm which originates from the wetting layer (WL). At longer wavelengths emission from single QDs is clearly observed extending to wavelengths ∼1000 nm. The brightest QD emission is found very close to the WL emission band, which is undesired as it prevents utilizing these for quantum applications, for example, single‐photon sources and quantum memories.

Figure 2b–d show similar μPL spectra collected from regions doped with 50 keV Sb at doses of 1E10 ions/cm^2^, 5E10 ions/cm^2^, and 1E11 ions/cm^2^ respectively. Integration of the WL PL band reveals that the intensity of this emission reduces with ion dose by ∼47%, ∼99%, and becoming almost fully quenched at the highest dose used. It is also observed the PL from QDs with emission wavelengths shorter than ∼900 nm are also quenched as the implanted Sb dose increases. At low implantation doses (up to 5E10 ions/cm^2^), a redshift in the ensemble PL is observed. Rather than indicating the onset of structural interdiffusion, this shift is attributed to preferential electronic quenching. In this regime, the high‐energy tail of the QD distribution, associated with the smaller dots (875–895 nm), is selectively suppressed. This occurs because carriers in smaller QDs experience a reduced effective confinement barrier (Δ*E*_*eff*_ = *E*_barrier_ – *E*_*QD*_), facilitating carrier leakage from higher‐lying excited states into nearby ion‐induced defects in the surrounding matrix [42]. The proximity to the WL barrier facilitates a higher probability of carrier escape via tunneling or local thermal activation, even at low temperatures [43]. The quenching sensitivity of the smaller QDs under non‐resonant excitation is driven by the interruption of the carrier recapture cycle. Although carriers in deeply confined (larger) dots remain localized, those in smaller dots can be re‐emitted into the Wetting Layer or barrier continuum. In a pristine lattice, these carriers are typically recaptured; however, the high density of FIB‐induced vacancies created near the QD/barrier interface provides a nonradiative pathway that intercepts these leaking carriers.

This leaves a set of QDs displaying emission redshifted from the WL PL as can be seen by the comparison of average wavelength with increasing dose in Figure 3a. This would lead them to be suitable for use in PIC and quantum applications. However, the emission intensity of QDs within this region is generally reduced with increasing ion dose in comparison to the unimplanted reference region.

**FIGURE 3:**
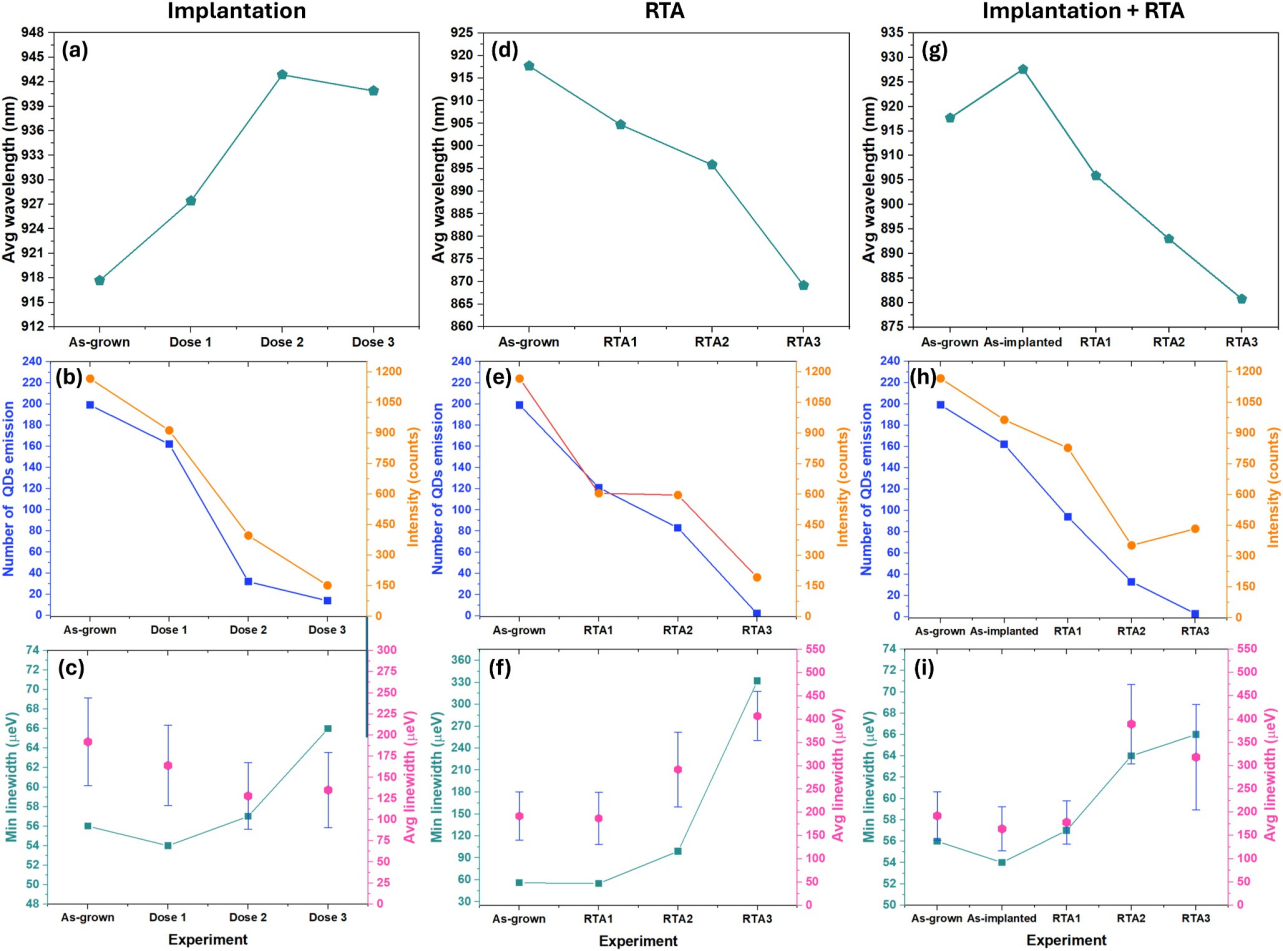
Effects of postgrowth engineering on InAs QD properties: (a–c) by Sb FIB implantation‐only (doses 1, 2, and 3 correspond to 1E10 ions/cm^2^, 5E10 ions/cm^2^, and 1E11 ions/cm^2^, respectively); (d–f) by RTA‐only (RTA1, 2, and 3 corresponds to 600°C, 700°C, and 800°C, respectively, all for 30 s); (g–i) by FIB implantation (Sb 50 keV and dose, 11E10 ions/cm^2^) and subsequent RTA.

The effect of RTA at temperatures from 600°C to 800°C on the PL of the as‐grown and Sb‐implanted InAs QDs is shown in Figure 4. The first observation is a blueshift in the ensemble emission that occurs for both unimplanted and implanted QDs. Atomic interdiffusion of Ga and In between the dots and barrier layers has been reported as responsible for such a blueshift which also affects the WL causing a slight wavelength shift [39]. Furthermore, non‐Fickian strain‐enhanced interdiffusion could play a role [44]. The strain in surrounding areas of the dots [45] promotes the vacancy concentration, leading to increase in the lateral group III interdiffusion.

**FIGURE 4:**
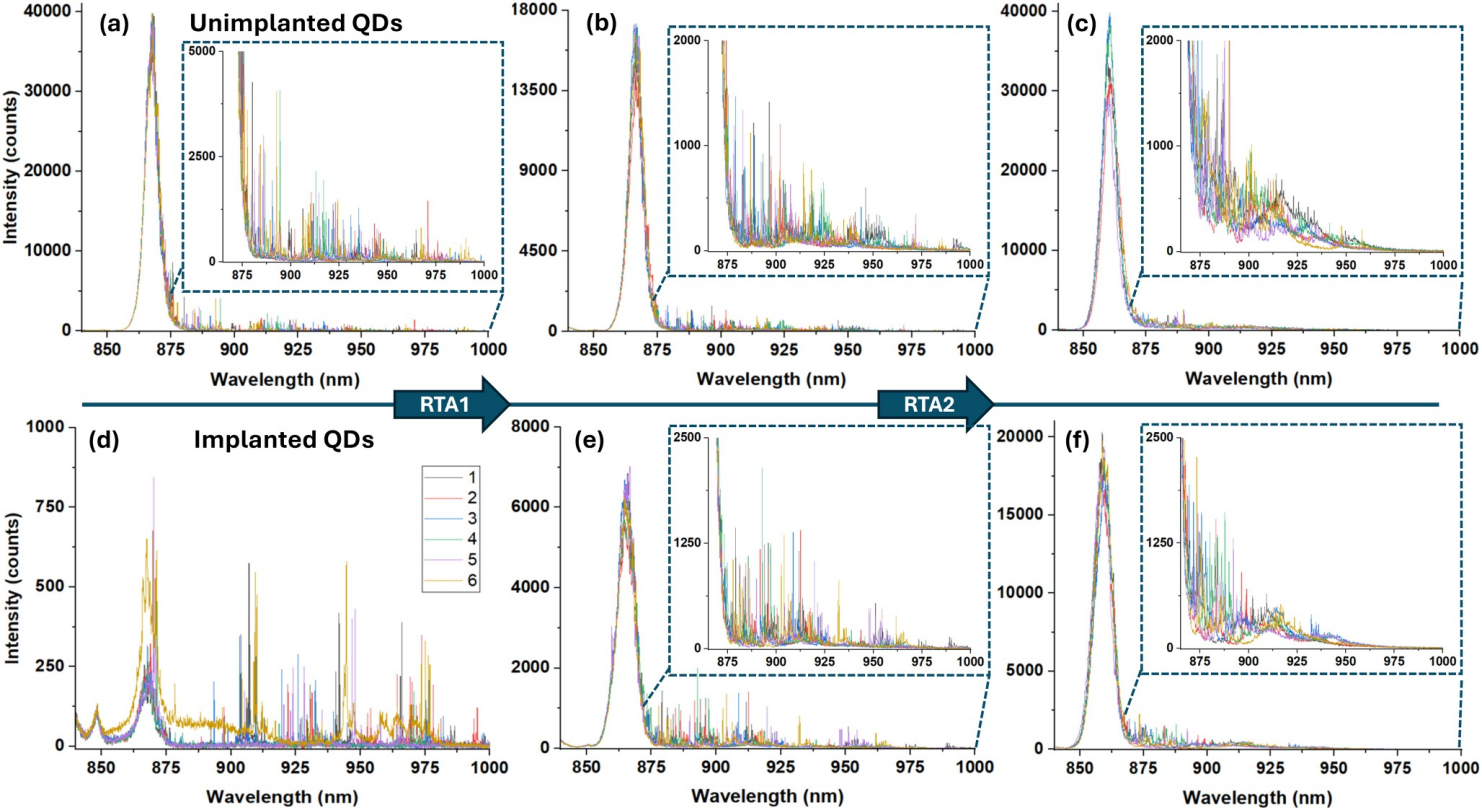
Effect of rapid thermal annealing on unimplanted QDs (top row, a–c) and implanted (Sb—50 keV – 1E10 ions/cm^2^) QDs (bottom row, d–f). RTA1 and RTA2 correspond to 600°C and 700°C, respectively, both for 30 s.

The wavelength shift increases with RTA temperature due to enhanced intermixing, but the Sb‐implanted QDs show larger blueshift compared to as‐grown dots in the same RTA conditions. This is consistent with the presence of extra vacancies created by ion implantation promoting intermixing during annealing.

During the RTA process, it has been observed that the QD shape evolves, becoming widened and tending to form elongated structures [37]. For example, an increase in InAs/GaAs QD lateral size has been reported due to interdiffusion [39]. However, at high temperatures, such as 800°C, the number of sharp lines originating from single dot emissions in the μPL spectra decreases drastically and a broad emission feature between 900 and 1000 nm appears. This transformation in the spectra indicates that the quantum confinement of the dots has been diminished. This is in agreement with previous TEM investigation where a complete dissolution of QDs into the WL was observed following RTA [39]. Strain‐enhanced lateral In/Ga interdiffusion could be responsible for the fast disappearance of the QDs in the investigated samples.

In addition to the integrated intensity, emission wavelength, and the number of QD emission lines observed, we studied the linewidth of the single QD emission in our high resolution μPL spectroscopy setup. The effect of different Sb FIB implantation and RTA conditions are summarized in Figure 3. Figure 3d,g show that the ensemble emission blueshift is observed whenever RTA is performed, whilst implantation alone is able to yield a redshift (Figure 3a). This redshift increases for implanted doses up to 5E10 ions/cm^2^ before saturating and is accompanied by a decrease in the average individual QD linewidth of ∼33% from 192 to 128 μeV (Figure 3c). Interestingly, although the average linewidth of QDs emission reduces with implantation, the minimum linewidth found within the ensemble actually increases (Figure 3b). There is a direct correlation between the number of individual QD emission lines observed and the integrated emission intensity.

The increase in wavelength shift and the decrease in linewidth saturate at an ion dose of ∼5E10 ions/cm^2^. These results show that by using optimal parameters a very good level of optical quality of QDs can be persevered after the implantation even in the case of here using heavy ion species (Sb). This is observed in Figure 3h where the integrated PL intensity for as‐implanted QDs is higher relative to annealed QDs. Moreover, the number of sharp emission lines of QDs can be higher than RTA‐based QDs (with or without implantation) as is evident in Figure 3h. Determination of the ideal parameters for implantation conditions is vital as other dose ranges will either create no change in QD properties or completely quench QD emission.

To explore the generality of the ion implantation approach to QD modification and the associated impact on optical properties, the study has been repeated using B implantation. B was selected due to it providing contrast to Sb both in terms of electronic doping (group III vs. group V) and mass (10.811 μ vs. 121.760 μ). To obtain a suitable ion energy for an appropriate ion penetration depth, similar modeling was performed for B ions (Supporting Information S1: Figure S1) resulting in 10 keV being chosen. Study of the QD μPL (Supporting Information S1: Figure S3) reveals similar trends to those observed following Sb implantation and are summarized in Figure 5. A redshift of the QD ensemble emission is observed, increasing with B‐dose, followed by a blueshift upon RTA. The average linewidth of the individual QD emission also decreases with B dose as previously observed for Sb implantation, and similarly the number of emitting QDs and the emission intensity is reduced. This indicates that this maskless postgrowth tailoring mechanism is versatile and reproducible and does not rely on specific elemental group or ion species making it applicable in different scenarios.

**FIGURE 5:**
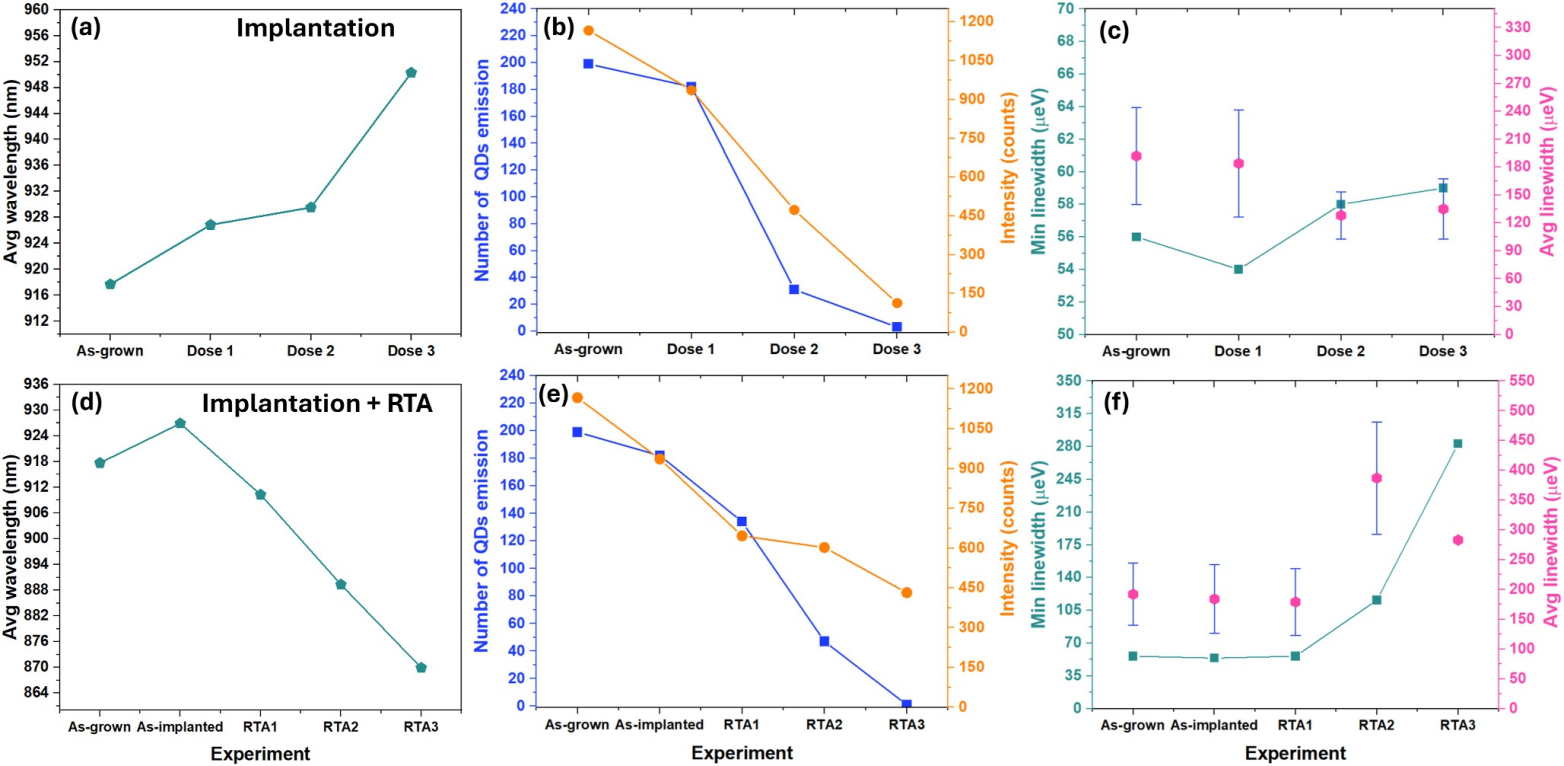
Effects of postgrowth engineering on InAs QD properties: (a–c) by Boron FIB implantation‐only (doses 1, 2, and 3 correspond to 1E10 ions/cm^2^, 1E11 ions/cm^2^, and 1E12 ions/cm^2^, respectively); (d–f) by RTA‐only (d–f) by FIB implantation (B 10 keV 1E10 ions/cm^2^) and subsequent RTA. RTA1, 2, and 3 corresponds to 600°C, 700°C, and 800°C, respectively, all for 30 s. Note that RTA‐only results were presented in Figure 3(d–f).

Through the utilization of different implantation and RTA conditions, it is possible to directly influence the emission characteristics of the QDs. Figure 6a clearly demonstrates the ability to adjust the ensemble average emission wavelength of QDs over a 70 nm range either toward shorter or longer wavelengths. Although blueshifts of 6.5 and 34 nm have been previous demonstrated by RTA‐only experiments [37], there is no direct report showing redshift by FIB implantation. Most works related to ion implantation technique are strongly reliant on subsequent annealing to recover QD emission [46, 47]. In addition to tailoring emission wavelength, Figure 6b shows how the implantation and RTA parameters affect the linewidth of tailored QDs. The associated results for B‐implanted QDs are also presented in Figure 6 which is generally consistent with the Sb‐implanted dots. Although slight variations in values are observed, likely resulting from differences in implantation‐induced damage, these can be mitigated through further optimization. This tailoring capability offers a range of tunable photonic properties for the InAs QDs that can be achieved for any specific integrated optoelectronic, nanophotonic, and quantum applications by postgrowth engineering technique.

**FIGURE 6:**
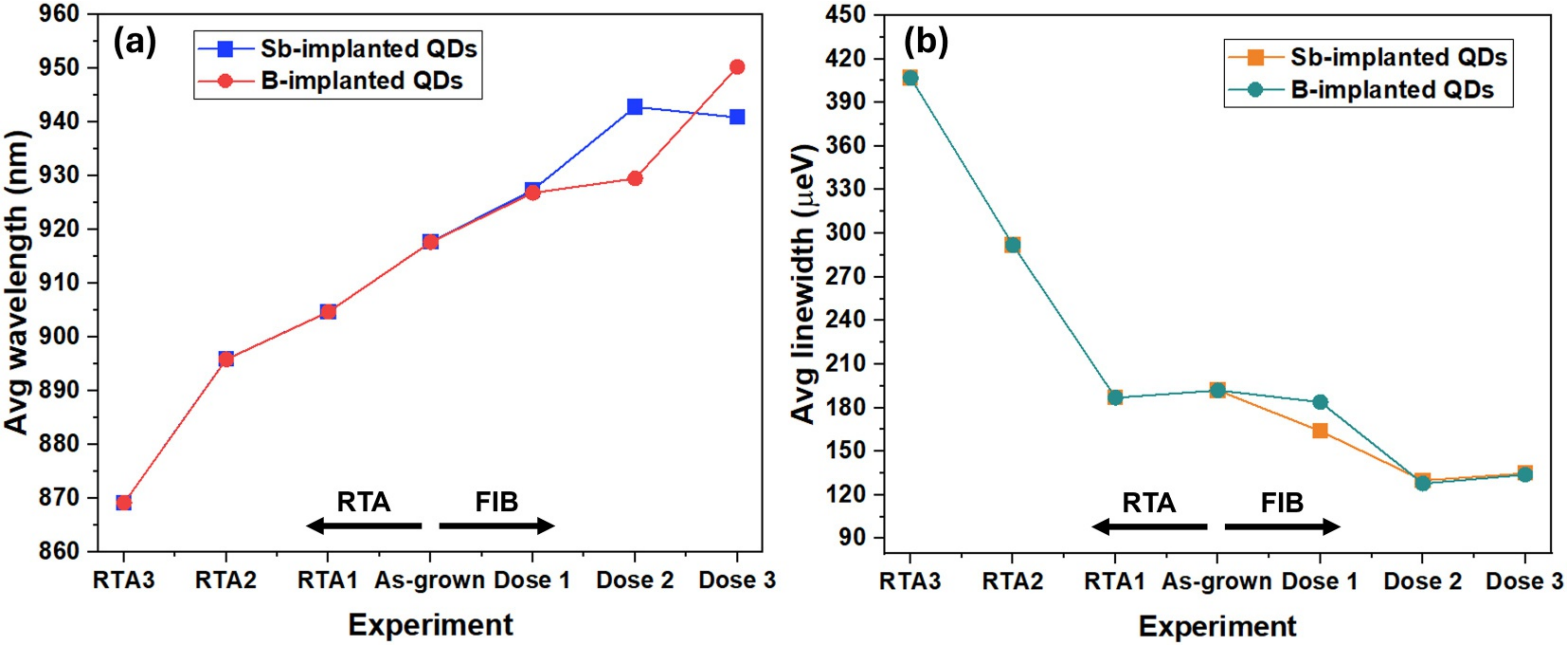
Tuning QDs' photonic properties (emission wavelength (a) and linewidth (b)) by single‐step postgrowth engineering experiments: comparison of effects of group III (B), group V (Sb) FIB implantation and RTA into the same QD sample. Single step means only one postgrowth process was used, either ion implantation or RTA. RTA1, 2, and 3 corresponds to 600°C, 700°C, and 800°C, respectively, all for 30 s. Doses 1, 2, and 3 correspond to 1E10 ions/cm^2^, 5E10 ions/cm^2^, and 1E11 ions/cm^2^ for Sb implantation and 1E10 ions/cm^2^, 1E11 ions/cm^2^, and 1E12 ions/cm^2^ for B implantation, respectively.

In general, ion implantation is able to change the bandgap, energy states, quantum confinement, and collection efficiency in low‐dimensional structures by modifying interfaces, elemental composition, defects, and strain fields [48, 49]. However, here the main mechanism is the selective suppression of QDs emitting at wavelengths close to the WL emission wavelength. This causes the redshift in the ensemble emission spectra of the retained QDs within the implanted region. In addition, this filtering mechanism by implantation affects inhomogeneous broadening which is an inherent critical drawback of epitaxial QD ensembles. This undesired phenomenon is the caused by the variation in QDs size, shape, and spatial distribution resulting in broadening of the ensemble emission linewidth. By the selective suppressing of lower wavelength emissions which are likely from the smaller dots, the inhomogeneous broadening (for ensemble) is mitigated (Figure 3c). We clarify that the narrowing of the PL ensemble is a result of ensemble trimming, where the ‘blue’ emitters are selectively filtered out (controllable by implantation parameters). Furthermore, changes in single‐QD linewidths postprocessing are attributed to the reconfiguration of the local electrostatic environment. The introduction of ion‐induced defects and their subsequent thermal passivation during RTA modifies the charge‐trap density in the QD vicinity, directly influencing spectral diffusion. A definitive study of these coherence properties is the subject of ongoing research [50]. Another promising point is that this filtering mechanism is tunable by implantation parameters (Figure 3a–c). It means one can tailor the PL suppression window, emission wavelength shift value, and number of QD (sharp) emission lines by selection of appropriate ion dose, for a specific target application. All these tailoring capabilities is achieved with site‐selectively and using a single‐step process of maskless FIB implantation without requiring subsequent annealing. This observation is noteworthy not only for postgrowth engineering of InAs QDs within this spectral range which is attractive for solid‐state quantum memory applications [24, 25] but also for separation of WL and QDs emission. This presents an entirely postgrowth solution to the mixing issue [51] that unlocks new opportunities for full utilization of InAs QDs in this wavelength range.

From implantation modeling results considering dynamic composition variations (Supporting Information S1: Figure S2), a higher dose significantly reduces the steepness of band structure between different layers which means weakening the quantum confinement. Also, the implantation damage is more pronounced in higher ion doses, resulting in reduced crystalline quality of the capping layer, barrier layer, and QDs. As a result, both quantum confinement and carrier collection efficiency are decreased within the structure leading to weaker emission. This explains why ion doses on the order of 1E12 ions/cm^2^ and above result in complete quenching of the emission from WL and QDs.

Notably, the presence of a single QD layer without subsequent thermal annealing ensures that these results reflect the direct effects of Sb FIB implantation on InAs QDs, in isolation from interlayer interactions, strain coupling, or other phenomena typically observed in multilayer structures.

## 5 Comparative Study

Finally, Table 1 presents a comparison of the work presented with previous works in the field of postgrowth engineering of QDs using ion implantation. This is the first time both red and blue shifts at the level of single dot emission are reported for epitaxial InAs QDs using FIB implantation‐based postgrowth direct engineering by a heavy group V element (Sb). Furthermore, in contrast to previous works in this field, our method ensures a high degree of area selectivity on the same QD sample without needing any mask or dielectric layer.

**TABLE 1:** Comparison of current work with the ion implantation‐based postgrowth engineering of InAs QDs reports in literature.

Ref	Implantation parameters	Maskless site‐selective process?	Single layer of QDs?	As‐implanted QD emission	Single dot emission resolution?	Average wavelength shift type	
This work	Sb—50 keV	Yes	Yes	Yes	Yes	Blue	Red
[52]	B—140 keV	No	No	No	No	Blue	—
[46]	B—120 keV	No	No	No	No	Blue	—
[46]	AR—120 keV	No	No	No	No	Blue	—
[47]	H—18 keV	No	Yes	No	No	Blue	—
[38]	H—40 keV	No	Yes	No	No	Blue	—
[53]	Mn—50 keV	No	Yes	Yes	No	Blue	—
[54]	P—450 keV	No	Yes	No	No	Blue	—
[55]	As—600 keV	No	Yes	Yes	No	Blue	—

Although conventional intermixing often utilizes thick capping layers (> 300 nm) and high‐energy ions, this approach results in a broad diffuse damage profile with ion straggle exceeding 100 nm (in different directions). In contrast, our shallow‐cap regime (45 nm) allows for low‐energy 50 keV Sb implantation, which constrains the depth straggle to < 10 nm and lateral straggle to ∼5 nm. This not only enables higher spatial resolution but also minimizes the total defect burden throughout the capping layer, explaining why optical signal is preserved at low doses despite the proximity to the surface. This gives substantial gain in 3D spatial accuracy, making this method suitable for the high‐density integration required in quantum photonic circuits. It enables us to target individual QDs or nanophotonic components without affecting the surrounding matrix. A quantitative comparison of ion straggle and ion‐induced damage for implantation parameters used in this work versus conventional works is provided in Supporting Information S1: Figure S4.

## 6 Conclusion and Perspectives

Finally, we have demonstrated a postgrowth maskless site‐selective method to tailor the photonic properties of InAs epitaxial QDs using FIB implantation. In contrast to previous studies that relied on postimplantation annealing, our approach preserved sharp QD emission lines without thermal treatment. Although RTA induced blueshifts as expected, FIB‐only implantation resulted in an ensemble redshift and reduced inhomogeneous broadening attributed to a filtering effect that suppresses emission from QDs near the wetting layer band.

This strategy not only addresses inhomogeneous broadening and spectral mixing issues with WL emission in short‐wavelength regimes but also breaks the conventional blueshift‐only limitation of post‐growth modification in such epitaxial nanostructures. Notably, the successful use of heavy Sb ions, reported here for the first time, demonstrates the feasibility of employing group V elements for precise, nondestructive photonic modification. Complementary experiments with light B ions confirmed the generality of the method across ion species. These group III/V elements is two‐folded important for photonic engineering of epitaxial QDs. Firstly, it evidences the feasibility of utilizing heavier elements to tailor QD properties without destroying the QDs by the process in the case of a well‐designed implantation strategy. Secondly, group III/V elements represents strategically valuable species for doping semiconductor structures.

Although this study establishes the ensemble‐level response to localized FIB implantation, future work will utilize high‐resolution single‐dot spectroscopy to quantify the impact of ion‐induced defects on homogeneous linewidths and spectral diffusion, leveraging established protocols for charge‐state control and Stark tuning recently demonstrated for individual emitters [50].

The spatial resolution and maskless nature of the FIB process provide a unique platform for site‐selective engineering. This direct‐write capability is particularly suited for the deterministic alignment of QDs with photonic crystal cavities. Future optimization of radiative efficiency through surface passivation and refined annealing protocols will further enhance the practicality of this method for integrated quantum photonic devices. Looking ahead, this work paves the way for postgrowth site‐selective single‐QD engineering via high‐resolution SEM‐integrated FIB implantation [31] and opens promising directions for quantum photonic device integration and postgrowth local doping of epitaxial QDs.

## Author Contributions

**Mehdi Ahmadian:** conceptualization, methodology, data curation, investigation, formal analysis, writing – original draft, writing – review and editing. **Young In Na:** methodology, investigation, formal analysis, writing – review and editing. **Maddison Coke:** investigation, writing – review and editing, methodology, formal analysis. **Daniel Blight:** investigation, writing – review and editing, methodology, formal analysis. **Akshay Kumar Verma:** methodology, investigation, formal analysis, writing – review and editing. **Mark C. Rosamond:** methodology, investigation, writing – review and editing. **Ian Farrer:** methodology, formal analysis, investigation, writing – review and editing. **Jon Heffernan:** conceptualization, methodology, supervision, funding acquisition, writing – review and editing, resources. **Richard J. Curry:** resources, supervision, conceptualization, investigation, funding acquisition, writing – review and editing.

## Funding

This work was supported by UK Engineering and Physical Sciences Research Council (EP/S024441/1, EP/X015300/1, EP/R025576/1, and EP/V001914/1).

## Conflicts of Interest

The authors declare no conflicts of interest.

## Supporting information


Supporting Information S1

## Data Availability

The data that support the findings of this study are available in the Supporting Information accompanying this article.
