# Supplementary material for: Photonic Properties of InAs Quantum Dots by Post‐Growth Maskless Site‐Selective Focused Ion Beam Implantation
Source: Nanophotonics. 2026 Jun 13;15(12):e70162. doi: 10.1002/nap2.70162 (PMC13642057; doi:10.1002/nap2.70162)
Supplement: Supplementary file 1 [file NAP2-15-e70162-s001.docx]

**Supplementary data:**

Photonic Properties of InAs Quantum Dots by Post-Growth Maskless Site-Selective Focused Ion Beam Implantation

Mehdi Ahmadian, Young In Na, Maddison Coke, Daniel Blight, Akshay Kumar Verma, Mark C Rosamond, Ian Farrer, Jon Heffernan, Richard J Curry*

M. Ahmadian, M. Coke, D. Blight, R.J. Curry

Photon Science Institute (PSI), Department of Electrical & Electronic Engineering, University of Manchester, Manchester, UK

E-mail: [richard.curry@manchester.ac.uk](mailto:richard.curry@manchester.ac.uk)

M. Ahmadian

EPSRC Centre for Doctoral Training (CDT) in Compound Semiconductor Manufacturing, Cardiff University, Cardiff, UK

Y. I. Na, A. K. Verma, I. Ferrer, J. Heffernan

National Epitaxy Facility (NEF), Nanoscience and Technology Centre, University of Sheffield, Sheffield, UK

M. C. Rosamond

School of Electronic and Electrical Engineering, University of Leeds, UK

**S1.** Simulation results for B Ion implantation into the QD structure.

**Figure S1.** Ion implantation simulation of B ion range (a) and induced vacancies (b) within structure as a function of ion energy.

**S2.** Simulation results for Sb Ion implantation into the QD structure.

**Figure S2.** Static and dynamic modelling results. a) Penetration depth and lateral range (main bars) along with ion straggle in both directions (error bars) for various beam energies. b) Eroded depth from surface due to implantation with beam energy of 50 keV (Sb) and different ion doses (considering dynamic composition variations induced by ion implantation). c,d) Atomic composition profiles within the structure after implantation with beam energy of 50 keV (Sb), doses of 1E12 ions/cm^2^ and 1E15 ions/cm^2^ (considering dynamic composition variations induced by ion implantation).

**S3.** Micro-PL spectroscopy results for B-implanted QDs

**Figure S3.** 635 nm excited μPL spectra from InAs QDs obtained at 5 K for: a) as-grown QDs, b) post-RTA at 600 °C for 30 sec, c-e) 10 keV B implanted with doses of 1E10 ions/cm^2^, 1E11 ions/cm^2^ and 1E12 ions/ cm^2^ respectively (without RTA) and f) QDs which were undergone FIB implantation (B 10 keV 1E10 ions/cm^2^) and subsequent RTA (600 °C for 30 sec). Each panel consists of 6 µPL spectra taken from each region numbered 6.

**S4.** Analysis of ion straggle in different directions and ion-induced damage for implantation parameters used in this work versus literature

**Figure S4.** Ion straggle (a) and implantation-induced damage (b) analysis into GaAs top capping layer for implantation parameters used in relevant works.
